# Comprehensive analysis and validation of autophagy-related gene in rheumatoid arthritis

**DOI:** 10.3389/fcell.2025.1563911

**Published:** 2025-03-20

**Authors:** Runrun Zhang, Wenhan Huang, Ting Zhao, Jintao Fang, Cen Chang, Dongyi He, Xinchang Wang

**Affiliations:** ^1^ The Second Clinical Medical College, Zhejiang Chinese Medical University, Hangzhou, China; ^2^ Department of Rheumatology, The Second Affiliated Hospital of Zhejiang Chinese Medical University, Hangzhou, China; ^3^ Guanghua Clinical Medical College, Shanghai University of Traditional Chinese Medicine, Shanghai, China; ^4^ Institute of Arthritis Research in Integrative Medicine, Shanghai Academy of Traditional Chinese Medicine, Shanghai, China

**Keywords:** rheumatoid arthritis, IRF4, autophagy, bioinformatics, machine learning

## Abstract

**Background:**

Rheumatoid arthritis (RA) is a chronic autoimmune disease in which autophagy is pivotal in its pathogenesis. This study aims to identify autophagy-related genes associated with RA and investigate their functional roles.

**Methods:**

We performed mRNA sequencing to identify differentially expressed genes (DEGs) between RA and osteoarthritis (OA) and intersected these with autophagy-related genes to obtain autophagy-related DEGs (ARDEGs) in RA. Bioinformatics and machine learning approaches were used to identify key biomarkers. Functional experiments, including real-time cellular analysis (RTCA), scratch healing, and flow cytometry, were conducted to examine the effects of gene silencing on the proliferation and migration of MH7A cells.

**Results:**

A total of 37 ARDEGs were identified in RA. Through bioinformatics analysis, interferon regulatory factor 4 (IRF4) emerged as a key hub gene, with its high expression confirmed in RA synovial tissues and RA FLS cells. IRF4 knockdown inhibited the proliferation and migration and promoted the death of MH7A cells.

**Conclusion:**

IRF4 is an autophagy-related diagnostic biomarker for RA. Targeting IRF4 could serve as a potential diagnostic and therapeutic strategy for RA, although further clinical studies are required to validate its effectiveness.

## Introduction

Rheumatoid arthritis (RA) is an autoimmune disease that impacts the joints, marked by the enlargement of the synovial membrane and bone degradation, along with the formation of new blood vessels and the infiltration of cells that promote inflammation (Tanaka et al., 2020). According to the spectrum of autoantibodies, RA can be classified as anti-citrullinated protein antibody (ACPA) negative or ACPA positive. The main pathological mechanisms of RA involve interactions between genetics, environment, metabolism, immunity, and microbial communities (Smolen et al., 2016; Finckh et al., 2022). RA affects 0.5%–1% of the global population. In addition to typical manifestations of joint destruction, RA often affects the skin, lungs, kidneys, and other organs (Koduri and Solomon, 2023; Park and Bathon, 2024). RA joint injury is an important cause of disability in the population, and as the disease progresses, the disability rate of RA patients continues to rise. Early diagnosis can prevent severe joint injuries and improve patient prognosis.

Autophagy is a process in which cells form a double-membrane structure (autophagosome) to engulf damaged organelles or abnormal proteins, which then fuse with lysosomes for degradation and recycling (Liu et al., 2023). It is a key mechanism by which cells respond to nutrient deprivation, damage, and stress (Rockel and Kapoor, 2016). Autophagy can be classified into different types based on its mechanisms and execution processes: macroautophagy, microautophagy, and chaperone-mediated autophagy (Fleming et al., 2022). and usually, macroautophagy is referred to as autophagy (Li et al., 2020). It is regulated by signaling pathways such as mTOR, AMPK, and PI3K/Akt (Cai et al., 2022; Yang et al., 2022). The expression of autophagy-related proteins (Beclin1, ATG5, LC3) in the synovial tissue of RA patients is significantly increased, and is significantly correlated with inflammatory markers (CRP, ESR) and autoantibodies (citrullinated peptide, rheumatoid factor) levels (Zhu et al., 2017). Furthermore, treatment with anti-TNF-α and IL-6R inhibitors has been shown to result in a reduction in autophagy levels (Chen et al., 2018). The role of autophagy in RA includes immune regulation, overactive synovial fibroblasts, production of inflammatory cytokines, and generation of osteoclasts (Zhao et al., 2021).

Interferon Regulatory Factor 4 (IRF4) is a key immune regulatory transcription factor that plays an important role in immune cell differentiation, inflammatory responses, and the regulation of autoimmunity (De Silva et al., 2012; Huber and Lohoff, 2014; Rodríguez-Carrio et al., 2019). In RA, IRF4 is an essential transcription factor for Th17 cell differentiation, regulating Th17 cell differentiation and the secretion of inflammatory cytokines (Biswas et al., 2010; van Hamburg and Tas, 2018); and the IRF4 gene single nucleotide polymorphisms are associated with RA susceptibility (López-Isac et al., 2016). Targeting IRF4 may offer new directions for RA treatment.

In this study, mRNA sequencing was performed on RA and OA synovial tissues to obtain the transcriptome profile of RA synovial tissues, and the intersection of autophagy-related genes was obtained to identify autophagy-related differentially expressed genes (ARDEGs) in RA. Subsequently, bioinformatics analysis combined with experimental verification was performed to identify autophagy-related biomarkers in RA. This is of great significance for elucidating the role of autophagy influenced by IRF4 in RA and identifying potential biomarkers for future RA research.

## Materials and methods

### Sample collection and mRNA sequencing

Synovial tissue samples were collected from 9 RA to 15 OA patients undergoing knee joint replacement at Shanghai Guanghua Hospital of Integrated Traditional Chinese and Western Medicine. The collected synovial tissue was divided into two parts: one part was frozen at −80°C for mRNA sequencing, and the other part was used to extract primary Fibroblast-like synoviocytes (FLS). The Ethics Committee of Guanghua Hospital approved the study (approval number: 2018-K-12). All patients provided written informed consent before the surgery. Brief patient information was included in Supplementary Material S1.

Total RNA extraction from the synovial tissue sample was performed with the Trizol reagent (Thermo Fisher, Waltham, MA, United States) per the manufacturer’s instructions. The libraries were constructed using the TruSeq Stranded mRNA LT Sample Prep Kit (Illumina, San Diego, CA, United States) by the manufacturer’s guidelines and then sequenced on the Illumina HiSeq X Ten platform.

### Identification of ARGs

Autophagy-related genes (ARGs) were downloaded from the Human Autophagy Database (http://autophagy.lu/) and the MsigDB Database (https://www.gsea-msigdb.org/gsea/msigdb).

### GSEA and GSVA of ARGs in RA

Gene Set Enrichment Analysis (GSEA) provides insights into the biological processes and pathways significantly enriched in the dataset, helping identify potential mechanisms underlying the observed phenotypic differences (Subramanian et al., 2005). The cut-off criteria indicating statistically significant differences were set as |NES|>1, P < 0.05, and FDR ≤0.25. The analysis was performed using the “clusterProfiler” package in R software (version 4.4.1), with the data set sourced from the Molecular Signatures Database v7.2 (MSigDB) (Liberzon et al., 2015).

Gene Set Variation Analysis (GSVA) is a nonparametric, unsupervised method to determine differences in enriched gene sets across various clusters (Hänzelmann et al., 2013). The “GSVA” package in R (version 4.4.1) was used to assign signal pathway variation scores to each gene set, assessing their biological roles. Gene sets were sourced from MSigDB. A significant change was defined by a |t value of the GSVA score| greater than 1.

### WGCNA of ARGs in RA

Weighted Gene Co-expression Network Analysis (WGCNA) was employed to construct a gene co-expression network and identify modules of highly correlated genes (Langfelder and Horvath, 2008). These hub genes are considered to play central roles in the biological processes represented by their respective modules. The analysis used the “WGCNA” package in R (version 4.4.1).

### Identification of ARDEGs

Differentially expressed genes (DEGs) were identified with the “DESeq” R package (Anders and Huber, 2012). Significant differential expression was determined with a threshold of adjust P-value <0.05 and |log2FoldChange| ≥ 1. The R package “VennDiagram” was used to plot a Venn diagram, overlapping DEGs with autophagy-related genes (ARGs), to obtain the autophagy-related DEGs (ARDEGs).

### Functional enrichment analysis of ARDEGs

The ARDEGs were annotated using Gene Ontology (GO) enrichment analysis, which included biological process (BP), cellular component (CC), molecular function (MF) (Gene Ontology Consortium, 2015), and Kyoto Encyclopedia of Genes and Genomes (KEGG) pathway analysis. KEGG is a database resource that helps elucidate molecular and higher-level gene functions, including biochemical pathways (Kanehisa et al., 2023). The annotation and visualization were performed with the “clusterProfiler” R package. A P-value <0.05 was used as the cut-off criterion to indicate statistical significance.

### The protein-protein interaction network construct and hub genes identified

The protein-protein interaction (PPI) network of ARDEGs was constructed using the STRING database, which can comprehensively describe user gene lists and functional genomic datasets and create and share highly customized and enhanced protein-protein association networks (Soleymani et al., 2022). The PPI network was visualized using the Cytoscape Plugin.

### Friends analysis for hub genes screening

Friends analysis is a method used to compare the similarities between different genes or gene sets. If a gene interacts with other genes in the pathway, then that gene may be more important, possibly known as a hub gene (Duan et al., 2022). It can help you select the most important genes from a pile of significantly differentially expressed genes.

### Hub genes identified based on multiple machine-learning methods

To further identify the candidate biomarkers, the least absolute shrinkage and selection operator (LASSO) algorithm, a logistic regression method for filtering variables to enhance the predictive performance (Wang et al., 2024), was adopted to screen the candidate genes with the “glmnet” R package.

Machine-learning predictive models include the support vector machine (SVM) and random forest (RF) models. SVM is a supervised learning model for classification and regression analysis. The basic idea of SVM is to find an optimal decision boundary that maximizes the margin between classes, thereby achieving data classification (Sollich, 2003). RF is a commonly used ensemble learning method for classification and regression tasks. It enhances the accuracy and robustness of the model by constructing multiple decision trees and combining their prediction results (Blanchet et al., 2020; Hu and Szymczak, 2023). The two machine learning models were explained using the “DALEX” R package, and residual distribution and feature importance among the models were visualized.

### Immunohistochemistry

The streptavidin-biotin peroxidase complex immunohistochemistry method was utilized for detection. Tissue sections were dewaxed using standard procedures and treated with 3% hydrogen peroxide (H_2_O_2_) for 10 min to block endogenous peroxidase activity. Three 5-min washes with PBS followed this. A blocking serum was applied to the sections for 20 min and removed. The primary antibody was incubated for 1 h, followed by another set of three 5-min washes in PBS. Streptavidin conjugated to horseradish peroxidase and biotin were applied for 20 min, with a subsequent 1-h incubation. Another series of three 5-min PBS washes were performed before applying DAB for 5 min to develop the staining. Finally, the sections were counterstained with hematoxylin for 2 min, rinsed in tap water, dehydrated, cleared, mounted, and examined under a microscope.

### Isolation of RA FLS and cell lines culture

Synovial tissues from the knee joint were placed in a 6 cm culture dish, washed twice with sterile PBS, and minced into small fragments using scissors. Based on tissue size, 2 mL of 4 mg/mL collagenase NB4 solution was added, and the samples were digested in a 37°C incubator with 5% CO_2_ for 2 h. Digestion was terminated by adding an equal volume of DMEM culture medium containing 10% FBS. The mixture was filtered through a 100 µm mesh and centrifuged at 1,500 rpm for 5 min. The supernatant was discarded, and the cell pellet was resuspended in 10% FBS DMEM. Cells were counted, inoculated into a 25 cm^2^ culture flask, and maintained in a 37°C, 5% CO_2_ incubator with medium changes 2–3 times per week. When the cells reach 70%–80% confluence, perform a 1:2 subculture. Cells at passages 4-5 are used for the next experiment.

Human rheumatoid arthritis fibroblast-like synoviocytes (MH7A) were obtained from Guangzhou Jennio Biotech Co., Ltd. These cells were cultured in DMEM supplemented with 10% FBS and 1% penicillin-streptomycin (P/S) and maintained in a 37°C incubator with 5% CO_2_.

### Quantitative real-time PCR

Total RNA was extracted with SteadyPure Quick RNA Extraction Kit (Accurate Biotechnology (Hunan) Co., Ltd.) and reverse-transcribed to complementary DNA (cDNA) with the Evo M-MLV RT Mix kit with gDNA Clean for qPCR Ver.2 (Accurate Biotechnology (Hunan) Co., Ltd.). The qRT-PCR was then performed using the SYBR Green Premix pro-Taq HS qPCR Kit (Accurate Biotechnology (Hunan) Co., Ltd.). GAPDH was used as the internal reference. The primers used were listed below:

**Table:** 

Species	Primer name	Sequence
Human	GAPDH forward	5′-GGAGCGAGATCCCTCCAAAAT-3′
	GAPDH reverse	5′-GGCTGTTGTCATACTTCTCATGG-3′
	IRF4 forward	5′-GCTGATCGACCAGATCGACAG-3′
	IRF4 reverse	5′-CGGTTGTAGTCCTGCTTGC-3′

The relative messenger RNA (mRNA) expression was calculated using the 2^−△△Ct^ method.

### Construction of IRF4 stable knockdown cell line

Lentivirus was constructed by Shanghai Xiangyou Technology Co., Ltd.

pLV-U6-shRNA1(IRF4)-CMV-EGFP-2A-Puro.

cgggcaagcaggactacaaccctcgagggttgtagtcctgcttgcccgttttt;

pLV-U6-shRNA2(IRF4)-CMV-EGFP-2A-Puro.

gtacaaagtgtacaggattgctcgagcaatcctgtacactttgtacgttttt;

pLV-U6-shRNA3(IRF4)-CMV-EGFP-2A-Puro.

gaagattaccacagatctatcctcgaggatagatctgtggtaatcttcttttt.

Preliminary experiments confirmed that shRNA1 (IRF4) had the highest infection efficiency and IRF4 MOI = 100. The puromycin screening concentration for MH7A cells was 4 μg/mL, and the subsequent experiments used 4 μg/mL to act on MH7A.

### Real-time cellular analysis (RTCA)

RTCA can be used for real-time dynamic cell viability detection without labeling. Inoculate 5 × 10^3^ cells into an E-plate16 well plate, culture the plate at 37°C, 5% CO_2_, and continuously detect cell viability using the xCELLigence system. The detection results are displayed in the form of a delta cell index.

### Scratch healing experiment

2 × 10^5^ cells were seeded into 6-well plates. After reaching 100% confluence, cells were gently scraped with a 200 μL tip, washed three times in serum-free medium, and cultured in a regular medium. Wound healing was observed at 0, 24 h, and 48 h, and the cell migration distance was calculated by subtracting the wound width at each time point from the wound width at 0 h. Three independent analyses were performed; images were collected at 0, 24, and 48 h after scratch formation, and the images were analyzed using ImageJ software.

### Calcein-AM/propidium iodide (PI) staining

Cell viability was detected by Meilun Calcein-AM/Pl Double Staining Kit (meilunbio). Cells were cultured for 48 h according to different treatments. Cells were collected and resuspended, and then 2 μM calcein AM and 8 μM PI were added to incubate for 15 min. The death rate was detected by flow cytometry.

### Western blot

Total protein was extracted from RA FLS, and its concentration was measured using a BCA kit (Beyotime). Protein samples were separated by sodium dodecyl sulfate-polyacrylamide gel electrophoresis (SDS-PAGE) and transferred onto PVDF membranes (Millipore). After blocking, the membranes were incubated with primary antibodies followed by horseradish peroxidase-conjugated secondary antibodies (Bio-Rad). Immunodetection was carried out using an enhanced chemiluminescence kit (Biosharp). Vaculin served as a loading control for normalization. The primary antibody used was an anti-IRF4 antibody (Proteintech). Bands were visualized using X-ray exposure with a Gel Imager (Bio-Rad).

### Statistical analysis

Data are expressed as mean ± standard deviation (SD) from at least three independent experiments. ANOVA test was performed using Prism 9.0 (GraphPad, San Diego, United States). P-values <0.05 were considered statistically significant. The data were presented as mean ± SD, and P < 0.05 was considered significant.

## Results

### The ARGs identified

**FIGURE 1:**
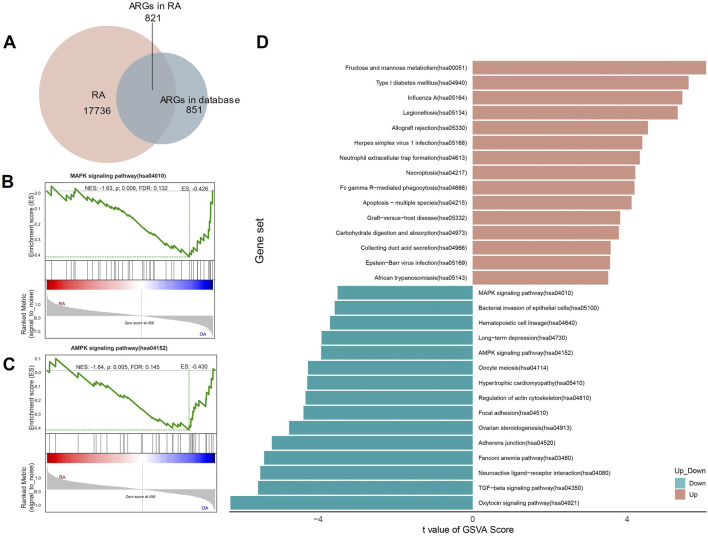
Autophagy-related genes (ARGs) identified and enrichment analyzed in RA. **(A)** The Venn Diagram of ARGs; **(B, C)** The GSEA of ARGs in RA; **(D)** The GSVA of ARGs in RA.

A total of 17,736 genes were detected in our mRNA sequencing data. 851 ARGs were extracted from the HADb and MSigDB databases. The two gene sets were intersected to obtain 821 ARGs in RA (Figure 1A).

### The GSEA and GSVA of ARGs in RA

To further investigate the functions related to ARGs, we performed GSEA and GSVA. The GSEA shows that for KEGG analysis, ARGs are rich in MAPK and AMPK signaling pathways (Figures 1B, C). The GSVA also showed that the MAPK and AMPK signaling pathways scored higher in the analysis (Figure 1D).

### The WGCNA of ARGs in RA

**FIGURE 2:**
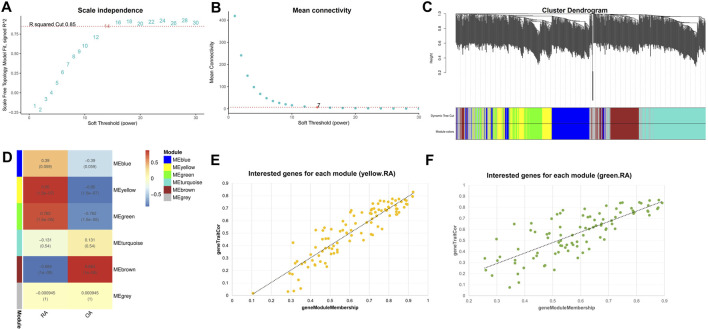
Construction of WGCNA modules. **(A)** Graph of scale independence; **(B)** Graph of mean connectivity; **(C)** Cluster dendrogram of the co-expression network modules; **(D)** Cluster plot analysis of the relationship between ARGs in RA and modules; **(E)** Scatter plot analysis of the yellow module; **(F)** Scatter plot analysis of the green module.

To identify key gene modules associated with RA, we utilized WGCNA. The soft-thresholding power was fourteen, determined based on an R-squared cut of 0.85 (Figures 2A, B). Six modules were identified based on average hierarchical clustering and dynamic tree clipping (Figure 2C). The yellow and green modules positively correlated with RA (Figure 2D). The yellow module screened 102 genes, and the green module screened 94 genes for subsequent analysis (Figures 2E, F).

### Identification of ARDEGs in RA

**FIGURE 3:**
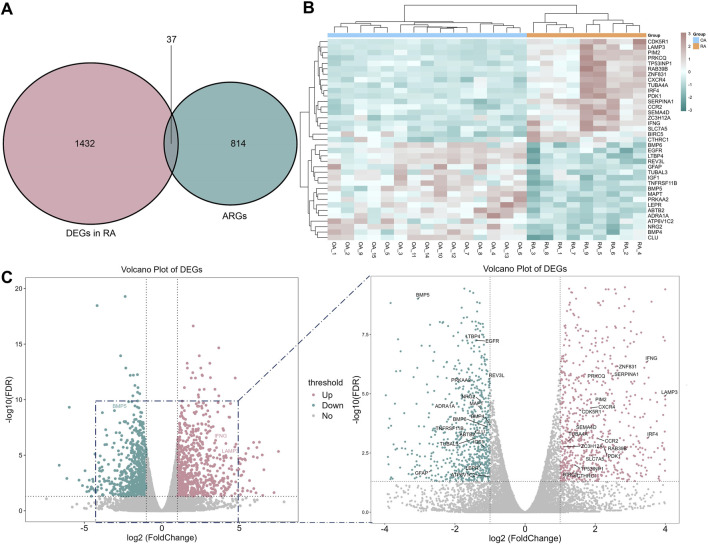
Autophagy-related DEGs (ARDEGs) identified. **(A)** The Venn diagram between autophagy and RA related genes; **(B)** The heat map of 37 related genes; **(C)** The volcano plot of ARDEGs.

The transcriptional profiles of RA patients exhibit distinct characteristics compared to OA patients, indicating a unique status in RA. Consequently, a differential expression analysis was conducted. With a threshold of adjusted P value <0.05 and |log2FoldChange| ≥ 1, 1,469 DEGs were identified and intersected with 851 ARGs, resulting in the identification of 37 ARDEGs (Figure 3A). The heat map of the 37 ARDEGs was shown in Figure 3B, and the volcano plot of ARDEGs (19 upregulated genes and 18 downregulated genes) was shown in Figure 3C.

### The GO and KEGG analysis of ARGDEGs

**FIGURE 4:**
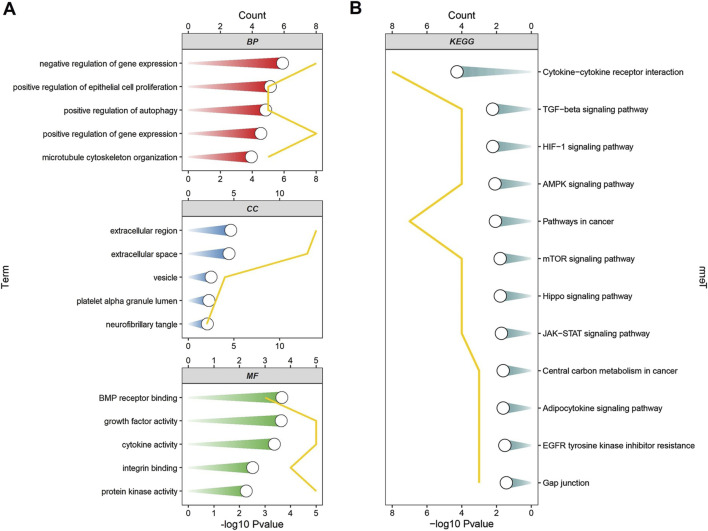
The enrichment analysis of ARDEGs. **(A)** GO functional enrichment analysis of ARDEGs; **(B)** KEGG pathway analysis of ARDEGs.

To further investigate the functions related to ARGDEGs, we performed GO and KEGG pathway. GO functional enrichment analysis showed that ARDEGs were mainly distributed in the negative regulation of gene expression, positive regulation of autophagy in BP, extracellular region, and extracellular space in CC. BMP receptor and cytokine activity in MF. (Figure 4A). KEGG pathway analysis showed that ARDEGs were enriched in the AMPK signaling pathway, mTOR signaling way, and cytokine-cytokine receptor interaction (Figure 4B).

### The PPI network analysis and hub genes screened

**FIGURE 5:**
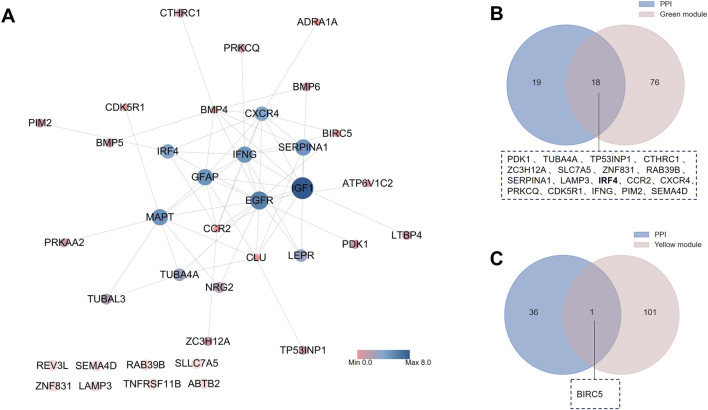
Identification of hub ARDEGs. **(A)** PPI network of ARDEGs; **(B)** Venn diagram of PPI and WGCNA green group; **(C)** Venn diagram of PPI and WGCNA green group.

With the STRING database, the network contained 37 nodes and 62 edges, and the enrichment P value was 2.12 × 10^−12^. The degree of every gene was obtained with the “Analyze Network” function of Cytoscape. Based on the degree, we constructed the PPI network (Figure 5A). We filtered hub genes based on the PPI network and WGCNA green module (Figure 5B) and yellow module (Figure 5C). These two modules are the gene sets identified by WGCNA as most relevant to RA. PDK1, TUBA4A, TP53INP1, CTHRC1, ZC3H12A, SLC7A5, ZNF831, RAB39B, SERPINA1, LAMP3, IRF4, CCR2, CXCR4, PRKCQ, CDK5R1, IFNG, PIM2, SEMA4D, BIRC5 were identified. With the top ten degrees from Cytoscape, the SERPINA1, IFNG, IRF4, CXCR4, and TUBA4A were defined as hub genes for the next analysis.

### Friends analysis and machine learning analysis

**FIGURE 6:**
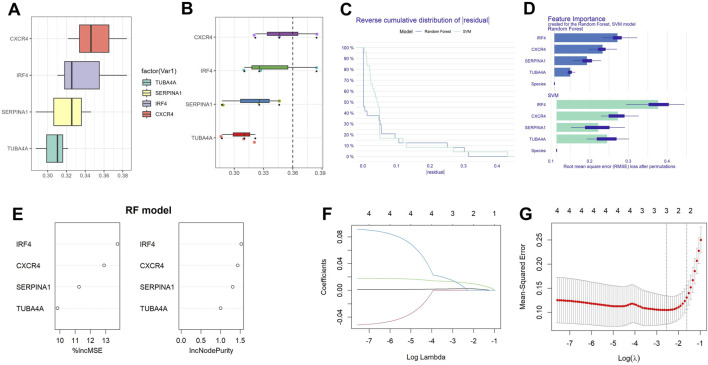
Establishment of diagnostic biomarkers. **(A)** Histogram of friends analysis of ARDEGs; **(B)** Cloud and rain map of friends analysis of ARDEGs; **(C)** Cumulative residual distribution of the two machine learning models; **(D)** Significant functions of the two machine learning models; **(E)** Variable importance plot of random forest model; **(F)** Regression coefficient convergence (path) diagram; **(G)** Cross validation diagram.

Further friendship analysis was adopted to analyze hub genes. According to its expression level in sequencing, IFNG has a relatively low expression level, so it is not included in further analysis. The top-ranked genes were CXCR4, IRF4, SERPINA1, and TUBA4A (Figures 6A, B). To further identify critical markers with high diagnostic values, we established two machine-learning models based on the blue core module expression profile, including SVM and RF models. The RF model had a relatively low residual (Figures 6C, D). With the RF model analysis, CXCR4 and IRF4 were identified as hub genes(Figure 6E). Using the LASSO regression algorithm, the hub genes were narrowed to 2 variables, CXCR4 and IRF4 (Figures 6F, G). CXCR5 has been reported to be associated with autophagy in RA (Huang et al., 2021), so IRF4 was chosen as the next research target.

### The expression of IRF4 in RA synovial tissue and FLSs

**FIGURE 7:**
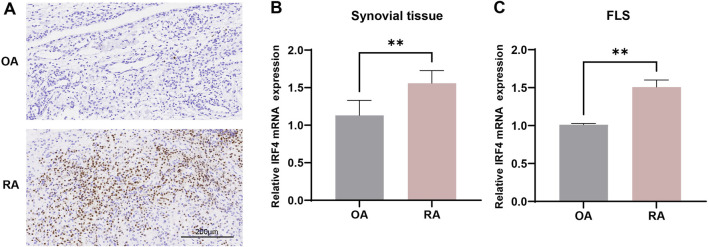
The expression of IRF4 in RA synovial tissues and cells. **(A)** The expression of IRF4 in RA and OA synovial tissues observed by immunohistochemistry (n = 4 in each group); **(B)** The expression of IRF4 in RA and OA synovial tissues observed by qRT-PCR (n = 3 in each group); **(C)** The expression of IRF4 in RA and OA synovial cells observed by qRT-PCR (n = 3 in each group). ** indicate p < 0.01.

Immunohistochemistry and qRT-PCR were used to validate the differential expression of IRF4 in RA and OA. Immunohistochemistry showed that IRF4 was highly expressed in RA synovial tissue (Figure 7A), while qRT-PCR revealed that IRF4 was upregulated in both RA synovial tissue (Figure 7B) and FLSs (Figure 7C).

### Effects of IRF4 silencing on MH7A cells

**FIGURE 8:**
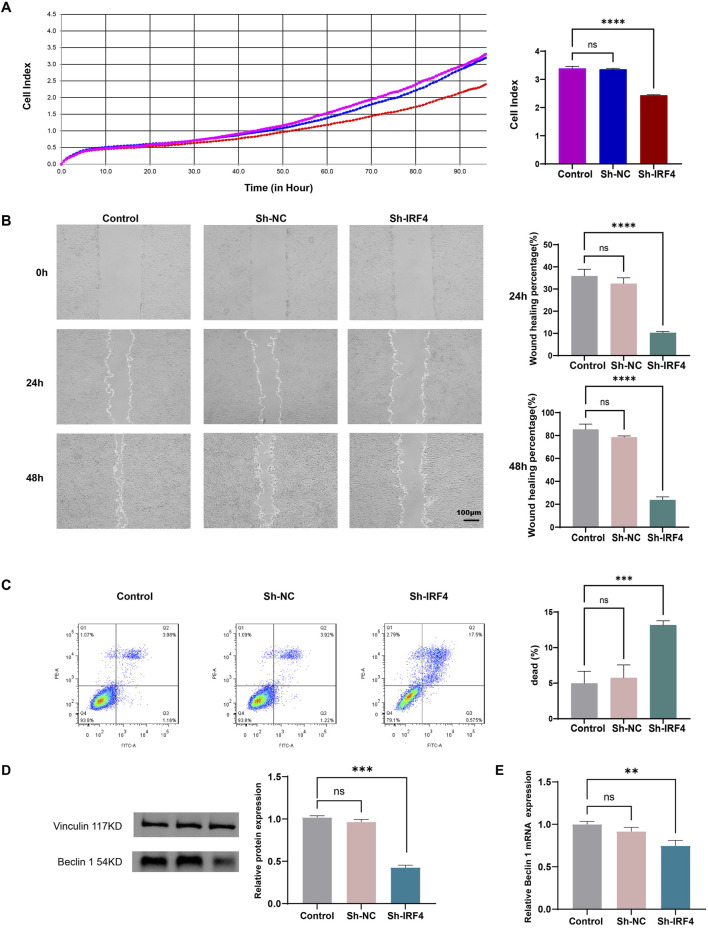
Effects of IRF4 silencing on MH7A. **(A)** Proliferative ability of MH7A with the above transfection, as determined by RTCA; **(B)** Migration of MH7A with the above transfection, as determined by scratch healing experiments; **(C)** Death of MH7A with the above transfection, as determined by flow cytometry; **(D)** The level of Beclin with the above transfection, as determined by WB; **(E)** The mRNA level of Beclin with the above transfection, as determined by qRT-PCR. The data were presented as mean ± SD. **, ***, **** indicate p < 0.01, p < 0.001, p < 0.0001, respectively. All experiments were repeated 3 times.

To determine the role of IRF4 in MH7A cells, we performed gene silencing of IRF4. The RTCA showed that knockdown of IRF4 inhibited the proliferation of RA MH7A cells (Figure 8A). Scratch healing experiment showed that the knockdown of IRF4 restrained the migration of MH7A cells (Figure 8B). Flow cytometry confirmed that silencing of IRF4 enhanced the death of MH7A cells (Figure 8C). Knockdown of IRF4 decreased the protein level of beclin 1 (Figure 8D), and decreased the mRNA expression of beclin1 (Figure 8E).

## Discussion

Autophagy is a core molecular pathway for the preservation of cellular and organismal homeostasis (Li et al., 2020). Dysfunction of autophagy is associated with various cancers, as well as cardiovascular, neurodegenerative, metabolic, pulmonary, renal, infectious, musculoskeletal, and ocular diseases (Mizushima et al., 2008). Targeting autophagy may be a feasible therapeutic approach to combating diseases (Bourdenx et al., 2021; Dong et al., 2021). Different methods for treating various diseases through targeted autophagy have been discovered. In cancer, one treatment method is to induce autophagy and enhance its tumor suppressive properties. Based on the concept that autophagy is a protein degradation system used to maintain homeostasis in the body, it can also be inhibited to treat cancer (Ramesh et al., 2019). Similarly, autophagy also plays a complex and significant role in the pathogenesis of RA (a tumor-like disease) (Klionsky et al., 2021). It influences immune regulation, synovial fibroblast activity, cytokine production, and osteoclastogenesis, all of which are critical in the development and progression of RA (Zhao et al., 2021). Targeting autophagy pathways presents a promising avenue for novel therapeutic approaches in managing RA (Mueller et al., 2021). Further research is essential to fully understand the intricate relationship between autophagy and RA and to translate these findings into effective clinical interventions.

Autophagy influences the function of immune cells such as macrophages, dendritic cells, and T cells (Germic et al., 2019). In RA, dysregulated autophagy can lead to abnormal immune responses, contributing to inflammation and joint damage (Wang et al., 2020). Autophagy facilitates the presentation of antigens to immune cells, thereby influencing the adaptive immune response. Aberrant autophagy in dendritic cells can alter this process, exacerbating autoimmunity in RA (Ireland and Unanue, 2011). In RA, synovial fibroblasts become hyperactive, contributing to joint inflammation and destruction. Autophagy helps regulate the survival and function of these cells (Rockel and Kapoor, 2016). Dysregulated autophagy in synovial fibroblasts can lead to increased proliferation and resistance to apoptosis, promoting persistent inflammation and joint damage (Kim et al., 2017; Zhou et al., 2020). Autophagy modulates the production of inflammatory cytokines such as TNF-α, IL-1β, and IL-6 (An et al., 2018; Chen et al., 2022). These cytokines play crucial roles in the pathogenesis of RA. Disruption of autophagy pathways can lead to excessive cytokine production, enhancing inflammation. Autophagy affects the differentiation and function of osteoclasts, the cells responsible for bone resorption. In RA, increased osteoclast activity contributes to bone erosion (Lin et al., 2013). Proper regulation of autophagy in osteoclasts is essential for maintaining bone health, and its dysregulation can lead to enhanced bone degradation in RA (Lei et al., 2023).

Li et al. also have studied the ARGs in RA. They analyzed the GSE93272 dataset from the Gene Expression Omnibus database. LASSO, unsupervised clustering, and WGCNA were performed to identify ARGs strongly linked to RA. Additionally, they developed RF, SVM, generalized linear model, and extreme gradient boosting—based on the selected marker genes. Subsequently, they constructed a nomogram to differentiate between healthy individuals and RA patients. Finally, their findings were validated with five independent external RA datasets (Li et al., 2024). Our research differs from its focus. Firstly, our study screened ARGs in RA through mRNA sequencing combined with ARGs. GSEA and GSVA revealed that these genes could be enriched in the MAPK and AMPK signaling pathways. Subsequently, we conducted WGCNA analysis to identify hub genes. DEG analysis was performed to screen ARDEGs between RA and OA synovial tissues. Functional analysis revealed that ARDEGs were enriched in autophagy-related pathways such as mTOR signaling pathway and AMPK signaling pathway. Additionally, PPI combined with WGCNA was used to identify hub genes. Combining friends analysis and machine learning models with LASSO, CXCR4 and IRF4 were identified as the biomarker. The role of CXCR4 antagonists in RA has also been studied (Tamamura and Fujii, 2005; Han et al., 2025). Additionally, the relationship between CXCR4 and autophagy in RA has been reported (Huang et al., 2021). According to our literature search, the relationship between IRF4 and autophagy in RA has not been reported. Therefore, IRF4 was identified as a biomarker for further investigation. Finally, IRF4-related functions were conducted using RTCA, scratch healing experiment, and flow cytometry. It was found that knocking down IRF4 could inhibit the proliferation and migration of MH7A cells and promote the death of MH7A cells.

However, some areas in our study can be improved, such as increasing the clinical sample size and conducting additional experimental studies to confirm critical mechanisms, such as the downstream effects of autophagy. This analysis lays the foundation for exploring the impact of autophagy on RA and provides potential insights for clinical diagnosis and the development of new RA therapies.

## Data Availability

The datasets presented in this study can be found in online repositories. The names of the repository/repositories and accession number(s) can be found in the article/Supplementary Material.
