# Supplementary material for: Comprehensive analysis and validation of autophagy-related gene in rheumatoid arthritis
Source: Front Cell Dev Biol. 2025 Mar 20;13:1563911. doi: 10.3389/fcell.2025.1563911 (PMC11965638; doi:10.3389/fcell.2025.1563911)
Supplement: Supplementary file 1 [file DataSheet1.PDF]

Supplementary file 1 The sample information

	Age (year)	Gender	H/W (cm/kg)	ESR (mm/h)	CRP (mg/L)	RF-IGM (IU/mL)	RF-IGG (U/mL)	RF-IGA (U/mL)	Anti-CCP (RU/mL)
RA1	73	F	150/47	65	19.6	25.3	22.39	22.32	326.2
RA2	62	F	155/55	40	5.15	9.69	2.2	0.63	<20
RA3	61	F	163/62	16	6.28	<20	6.91	188.63	1600
RA4	57	F	150/35	80	178.3	528	198	300	1197.4
RA5	72	M	165/50	53	10.96	45.2	24.15	22.02	1555.9
RA6	70	F	150/45	27	20.68	/	/	/	/
RA7	70	F	160/71	48	<0.5	/	/	/	/
RA8	64	F	163/60	65	44.28	<10.1	0.15	0.26	20
RA9	75	F	160/55	66.76	72.52	/	/	/	/
OA1	75	F	155/63	10	0.62	83.8	43.02	300	1473
OA2	72	F	164/72	6	<0.5	/	/	/	/
OA3	85	F	160/65	17	0.7	/	/	/	/
OA4	83	F	156/67	31	1.82	/	/	/	/
OA5	74	F	146/57	9	0.63	/	/	/	/
OA6	69	F	16065	12	<0.5	/	/	/	/
OA7	71	F	156/67	15	1.89	/	/	/	/
OA8	67	F	160/65	4	<0.5	/	/	/	/
OA9	82	F	153/50	81	42.19	<10.10	0.36	0.64	20
OA10	68	F	158/54	12	0.66	/	/	/	/
OA11	68	F	160/46	17	<0.5	/	/	/	/
OA12	59	F	154/70	5	<0.5	/	/	/	/
OA13	75	F	158/52	3	<0.5	/	/	/	/
OA14	74	F	159/60	3	<0.5	/	/	/	/

OA15	73	F	155/55	10	0.7	/	/	/	/

H/W, Height/Weight; F, Female; M, Male; ESR, Erythrocyte Sedimentation Rate; CRP, C-reactive protein; RF, Rheumatoid Factor; CCP, Cyclic Citrullinated Peptide Antibody. Smoking: None of the patients, Hypertension: RA7 (10 years)/RA8 (20 years)/RA9 (5 years)/OA2 (20 years)/OA3 (10 years)/OA5 (5 years)/OA6 (10 years)/OA8 (10 years)/OA12(4 years), Diabetes: RA7 (3 years), OA12 (3 years).
